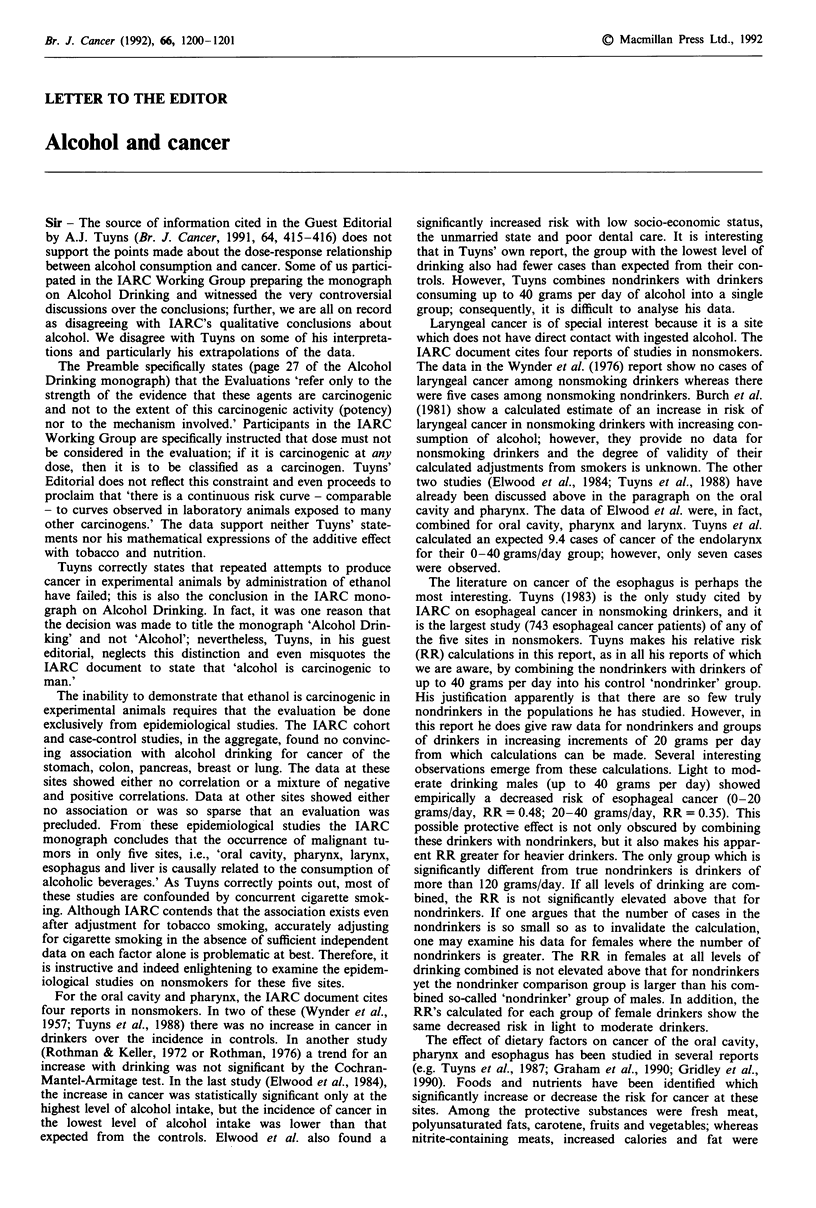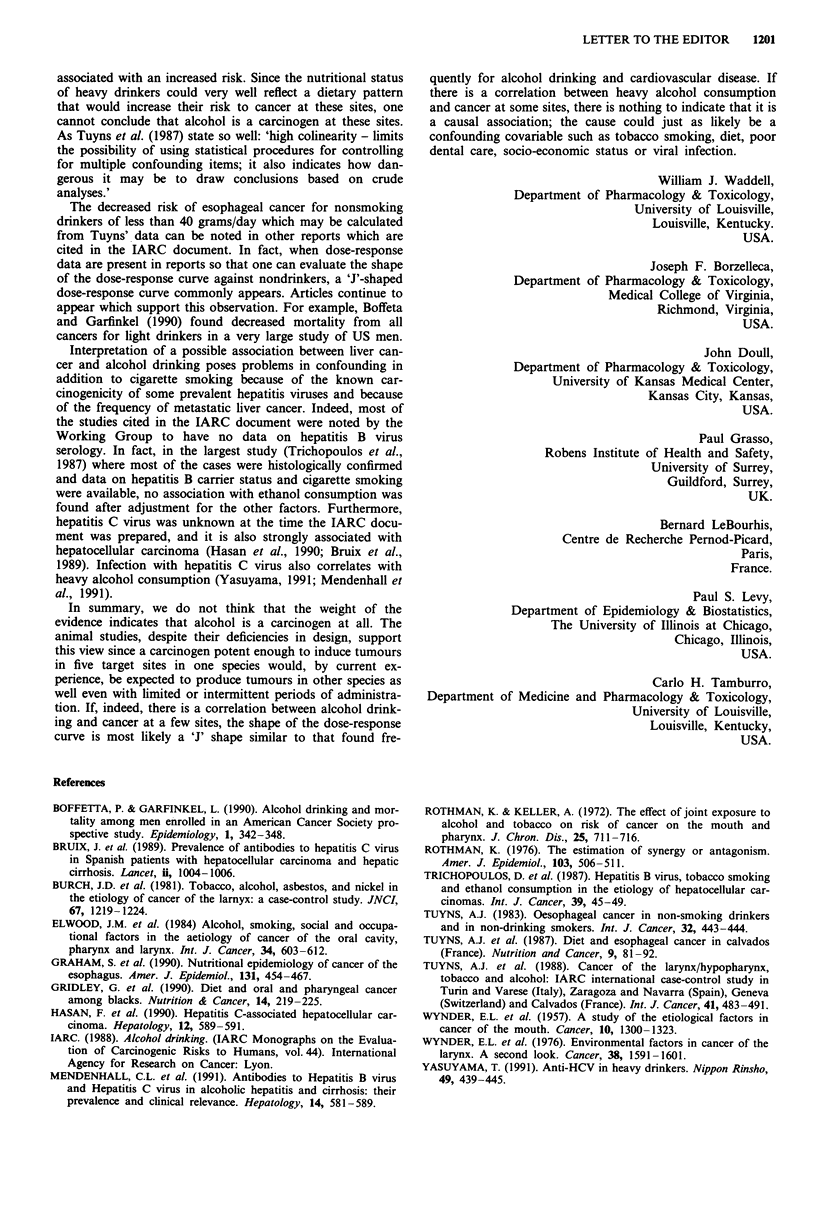# Alcohol and cancer.

**DOI:** 10.1038/bjc.1992.436

**Published:** 1992-12

**Authors:** W. J. Waddell, J. F. Borzelleca, J. Doull, P. Grasso, B. LeBourhis, P. S. Levy, C. H. Tamburro


Br. J. Cancer (1992), 66, 1200–1201
© Macmillan Press Ltd., 1992

LETTER TO THE EDITOR

### Alcohol and cancer

**Sir** – The source of information cited in the Guest Editorial by A.J. Tuyns (*Br. J. Cancer*, 1991, 64, 415–416) does not support the points made about the dose-response relationship between alcohol consumption and cancer. Some of us participated in the IARC Working Group preparing the monograph on Alcohol Drinking and witnessed the very controversial discussions over the conclusions; further, we are all on record as disagreeing with IARC's qualitative conclusions about alcohol. We disagree with Tuyns on some of his interpretations and particularly his extrapolations of the data.

The Preamble specifically states (page 27 of the Alcohol Drinking monograph) that the Evaluations 'refer only to the strength of the evidence that these agents are carcinogenic and not to the extent of this carcinogenic activity (potency) nor to the mechanism involved.' Participants in the IARC Working Group are specifically instructed that dose must not be considered in the evaluation; if it is carcinogenic at *any* dose, then it is to be classified as a carcinogen. Tuyns' Editorial does not reflect this constraint and even proceeds to proclaim that 'there is a continuous risk curve – comparable – to curves observed in laboratory animals exposed to many other carcinogens.' The data support neither Tuyns' statements nor his mathematical expressions of the additive effect with tobacco and nutrition.

Tuyns correctly states that repeated attempts to produce cancer in experimental animals by administration of ethanol have failed; this is also the conclusion in the IARC monograph on Alcohol Drinking. In fact, it was one reason that the decision was made to title the monograph 'Alcohol Drinking' and not 'Alcohol'; nevertheless, Tuyns, in his guest editorial, neglects this distinction and even misquotes the IARC document to state that 'alcohol is carcinogenic to man.'

The inability to demonstrate that ethanol is carcinogenic in experimental animals requires that the evaluation be done exclusively from epidemiological studies. The IARC cohort and case-control studies, in the aggregate, found no convincing association with alcohol drinking for cancer of the stomach, colon, pancreas, breast or lung. The data at these sites showed either no correlation or a mixture of negative and positive correlations. Data at other sites showed either no association or was so sparse that an evaluation was precluded. From these epidemiological studies the IARC monograph concludes that the occurrence of malignant tumors in only five sites, i.e., 'oral cavity, pharynx, larynx, esophagus and liver is causally related to the consumption of alcoholic beverages.' As Tuyns correctly points out, most of these studies are confounded by concurrent cigarette smoking. Although IARC contends that the association exists even after adjustment for tobacco smoking, accurately adjusting for cigarette smoking in the absence of sufficient independent data on each factor alone is problematic at best. Therefore, it is instructive and indeed enlightening to examine the epidemiological studies on nonsmokers for these five sites.

For the oral cavity and pharynx, the IARC document cites four reports in nonsmokers. In two of these (Wynder *et al.*, 1957; Tuyns *et al.*, 1988) there was no increase in cancer in drinkers over the incidence in controls. In another study (Rothman & Keller, 1972 or Rothman, 1976) a trend for an increase with drinking was not significant by the Cochran-Mantel-Armitage test. In the last study (Elwood *et al.*, 1984), the increase in cancer was statistically significant only at the highest level of alcohol intake, but the incidence of cancer in the lowest level of alcohol intake was lower than that expected from the controls. Elwood *et al.* also found a significantly increased risk with low socio-economic status, the unmarried state and poor dental care. It is interesting that in Tuyns' own report, the group with the lowest level of drinking also had fewer cases than expected from their controls. However, Tuyns combines nondrinkers with drinkers consuming up to 40 grams per day of alcohol into a single group; consequently, it is difficult to analyse his data.

Laryngeal cancer is of special interest because it is a site which does not have direct contact with ingested alcohol. The IARC document cites four reports of studies in nonsmokers. The data in the Wynder *et al.* (1976) report show no cases of laryngeal cancer among nonsmoking drinkers whereas there were five cases among nonsmoking nondrinkers. Burch *et al.* (1981) show a calculated estimate of an increase in risk of laryngeal cancer in nonsmoking drinkers with increasing consumption of alcohol; however, they provide no data for nonsmoking drinkers and the degree of validity of their calculated adjustments from smokers is unknown. The other two studies (Elwood *et al.*, 1984; Tuyns *et al.*, 1988) have already been discussed above in the paragraph on the oral cavity and pharynx. The data of Elwood *et al.* were, in fact, combined for oral cavity, pharynx and larynx. Tuyns *et al.* calculated an expected 9.4 cases of cancer of the endolarynx for their 0–40 grams/day group; however, only seven cases were observed.

The literature on cancer of the esophagus is perhaps the most interesting. Tuyns (1983) is the only study cited by IARC on esophageal cancer in nonsmoking drinkers, and it is the largest study (743 esophageal cancer patients) of any of the five sites in nonsmokers. Tuyns makes his relative risk (RR) calculations in this report, as in all his reports of which we are aware, by combining the nondrinkers with drinkers of up to 40 grams per day into his control 'nondrinker' group. His justification apparently is that there are so few truly nondrinkers in the populations he has studied. However, in this report he does give raw data for nondrinkers and groups of drinkers in increasing increments of 20 grams per day from which calculations can be made. Several interesting observations emerge from these calculations. Light to moderate drinking males (up to 40 grams per day) showed empirically a decreased risk of esophageal cancer (0–20 grams/day, RR = 0.48; 20–40 grams/day, RR = 0.35). This possible protective effect is not only obscured by combining these drinkers with nondrinkers, but it also makes his apparent RR greater for heavier drinkers. The only group which is significantly different from true nondrinkers is drinkers of more than 120 grams/day. If all levels of drinking are combined, the RR is not significantly elevated above that for nondrinkers. If one argues that the number of cases in the nondrinkers is so small so as to invalidate the calculation, one may examine his data for females where the number of nondrinkers is greater. The RR in females at all levels of drinking combined is not elevated above that for nondrinkers yet the nondrinker comparison group is larger than his combined so-called 'nondrinker' group of males. In addition, the RR's calculated for each group of female drinkers show the same decreased risk in light to moderate drinkers.

The effect of dietary factors on cancer of the oral cavity, pharynx and esophagus has been studied in several reports (e.g. Tuyns *et al.*, 1987; Graham *et al.*, 1990; Gridley *et al.*, 1990). Foods and nutrients have been identified which significantly increase or decrease the risk for cancer at these sites. Among the protective substances were fresh meat, polyunsaturated fats, carotene, fruits and vegetables; whereas nitrite-containing meats, increased calories and fat were associated with an increased risk. Since the nutritional status of heavy drinkers could very well reflect a dietary pattern that would increase their risk to cancer at these sites, one cannot conclude that alcohol is a carcinogen at these sites. As Tuyns *et al.* (1987) state so well: 'high colinearity – limits the possibility of using statistical procedures for controlling for multiple confounding items; it also indicates how dangerous it may be to draw conclusions based on crude analyses.'

The decreased risk of esophageal cancer for nonsmoking drinkers of less than 40 grams/day which may be calculated from Tuyns' data can be noted in other reports which are cited in the IARC document. In fact, when dose-response data are present in reports so that one can evaluate the shape of the dose-response curve against nondrinkers, a 'J'-shaped dose-response curve commonly appears. Articles continue to appear which support this observation. For example, Boffeta and Garfinkel (1990) found decreased mortality from all cancers for light drinkers in a very large study of US men.

Interpretation of a possible association between liver cancer and alcohol drinking poses problems in confounding in addition to cigarette smoking because of the known carcinogenicity of some prevalent hepatitis viruses and because of the frequency of metastatic liver cancer. Indeed, most of the studies cited in the IARC document were noted by the Working Group to have no data on hepatitis B virus serology. In fact, in the largest study (Trichopoulos *et al.*, 1987) where most of the cases were histologically confirmed and data on hepatitis B carrier status and cigarette smoking were available, no association with ethanol consumption was found after adjustment for the other factors. Furthermore, hepatitis C virus was unknown at the time the IARC document was prepared, and it is also strongly associated with hepatocellular carcinoma (Hasan *et al.*, 1990; Bruix *et al.*, 1989). Infection with hepatitis C virus also correlates with heavy alcohol consumption (Yasuyama, 1991; Mendenhall *et al.*, 1991).

In summary, we do not think that the weight of the evidence indicates that alcohol is a carcinogen at all. The animal studies, despite their deficiencies in design, support this view since a carcinogen potent enough to induce tumours in five target sites in one species would, by current experience, be expected to produce tumours in other species as well even with limited or intermittent periods of administration. If, indeed, there is a correlation between alcohol drinking and cancer at a few sites, the shape of the dose-response curve is most likely a 'J' shape similar to that found frequently for alcohol drinking and cardiovascular disease. If there is a correlation between heavy alcohol consumption and cancer at some sites, there is nothing to indicate that it is a causal association; the cause could just as likely be a confounding covariable such as tobacco smoking, diet, poor dental care, socio-economic status or viral infection.

William J. Waddell,
Department of Pharmacology & Toxicology,
University of Louisville,
Louisville, Kentucky.
USA.

Joseph F. Borzelleca,
Department of Pharmacology & Toxicology,
Medical College of Virginia,
Richmond, Virginia,
USA.

John Doull,
Department of Pharmacology & Toxicology,
University of Kansas Medical Center,
Kansas City, Kansas,
USA.

Paul Grasso,
Robens Institute of Health and Safety,
University of Surrey,
Guildford, Surrey,
UK.

Bernard LeBourhis,
Centre de Recherche Pernod-Picard,
Paris,
France.

Paul S. Levy,
Department of Epidemiology & Biostatistics,
The University of Illinois at Chicago,
Chicago, Illinois,
USA.

Carlo H. Tamburro,
Department of Medicine and Pharmacology & Toxicology,
University of Louisville,
Louisville, Kentucky,
USA.

### References

BOFFETTA, P. & GARFINKEL, L. (1990). Alcohol drinking and mortality among men enrolled in an American Cancer Society prospective study. *Epidemiology*, **1**, 342–348.

BRUIX, J. *et al.* (1989). Prevalence of antibodies to hepatitis C virus in Spanish patients with hepatocellular carcinoma and hepatic cirrhosis. *Lancet*, **ii**, 1004–1006.

BURCH, J.D. *et al.* (1981). Tobacco, alcohol, asbestos, and nickel in the etiology of cancer of the larnyx: a case-control study. *JNCI*, **67**, 1219–1224.

ELWOOD, J.M. *et al.* (1984) Alcohol, smoking, social and occupational factors in the aetiology of cancer of the oral cavity, pharynx and larynx. *Int. J. Cancer*, **34**, 603–612.

GRAHAM, S. *et al.* (1990). Nutritional epidemiology of cancer of the esophagus. *Amer. J. Epidemiol.*, **131**, 454–467.

GRIDLEY, G. *et al.* (1990). Diet and oral and pharyngeal cancer among blacks. *Nutrition & Cancer*, **14**, 219–225.

HASAN, F. *et al.* (1990). Hepatitis C-associated hepatocellular carcinoma. *Hepatology*, **12**, 589–591.

IARC. (1988). *Alcohol drinking.* (IARC Monographs on the Evaluation of Carcinogenic Risks to Humans, vol. 44). International Agency for Research on Cancer: Lyon.

MENDENHALL, C.L. *et al.* (1991). Antibodies to Hepatitis B virus and Hepatitis C virus in alcoholic hepatitis and cirrhosis: their prevalence and clinical relevance. *Hepatology*, **14**, 581–589.

ROTHMAN, K. & KELLER, A. (1972). The effect of joint exposure to alcohol and tobacco on risk of cancer on the mouth and pharynx. *J. Chron. Dis.*, **25**, 711–716.

ROTHMAN, K. (1976). The estimation of synergy or antagonism. *Amer. J. Epidemiol.*, **103**, 506–511.

TRICHOPOULOS, D. *et al.* (1987). Hepatitis B virus, tobacco smoking and ethanol consumption in the etiology of hepatocellular carcinomas. *Int. J. Cancer*, **39**, 45–49.

TUYNS, A.J. (1983). Oesophageal cancer in non-smoking drinkers and in non-drinking smokers. *Int. J. Cancer*, **32**, 443–444.

TUYNS, A.J. *et al.* (1987). Diet and esophageal cancer in calvados (France). *Nutrition and Cancer*, **9**, 81–92.

TUYNS, A.J. *et al.* (1988). Cancer of the larynx/hypopharynx, tobacco and alcohol: IARC international case-control study in Turin and Varese (Italy), Zaragoza and Navarra (Spain), Geneva (Switzerland) and Calvados (France). *Int. J. Cancer*, **41**, 483–491.

WYNDER, E.L. *et al.* (1957). A study of the etiological factors in cancer of the mouth. *Cancer*, **10**, 1300–1323.

WYNDER, E.L. *et al.* (1976). Environmental factors in cancer of the larynx. A second look. *Cancer*, **38**, 1591–1601.

YASUYAMA, T. (1991). Anti-HCV in heavy drinkers. *Nippon Rinsho*, **49**, 439–445.